# Identification of Phytoconstituents as Potent Inhibitors of Casein Kinase-1 Alpha Using Virtual Screening and Molecular Dynamics Simulations

**DOI:** 10.3390/pharmaceutics13122157

**Published:** 2021-12-15

**Authors:** Alaa Shafie, Shama Khan, Zehra, Taj Mohammad, Farah Anjum, Gulam Mustafa Hasan, Dharmendra Kumar Yadav, Md. Imtaiyaz Hassan

**Affiliations:** 1 Department of Clinical Laboratory Sciences, College of Applied Medical Sciences, Taif University, P.O. Box 11099, Taif 21944, Saudi Arabia; dr.alaa.shafie.tu@gmail.com (A.S.); farahanjum@tu.edu.sa (F.A.); 2 Drug Discovery and Development Centre (H3D), University of Cape Town, Rondebosch 7701, South Africa; shamak361@gmail.com; 3 Department of Computer Science, Jamia Millia Islamia, Jamia Nagar, New Delhi 110025, India; neelambeigh786@gmail.com; 4 Centre for Interdisciplinary Research in Basic Sciences, Jamia Millia Islamia, Jamia Nagar, New Delhi 110025, India; taj144796@st.jmi.ac.in; 5 Department of Biochemistry, College of Medicine, Prince Sattam Bin Abdulaziz University, Al-Kharj 11942, Saudi Arabia; mgulam@gmail.com; 6 College of Pharmacy, Gachon University of Medicine and Science, Hambakmoeiro, Yeonsu-gu, Incheon City 21924, Korea

**Keywords:** casein kinase-1 alpha, phytoconstituents, drug discovery, virtual screening, molecular dynamics simulation, dynamical cross-correlation matrices, principal components analysis

## Abstract

Casein kinase-1 alpha (CK1α) is a multifunctional protein kinase that belongs to the serine/threonine kinases of the CK1α family. It is involved in various signaling pathways associated with chromosome segregation, cell metabolism, cell cycle progression, apoptosis, autophagy, etc. It has been known to involve in the progression of many diseases, including cancer, neurodegeneration, obesity, and behavioral disorders. The elevated expression of CK1α in diseased conditions facilitates its selective targeting for therapeutic management. Here, we have performed virtual screening of phytoconstituents from the IMPPAT database seeking potential inhibitors of CK1α. First, a cluster of compounds was retrieved based on physicochemical parameters following Lipinski’s rules and PAINS filter. Further, high-affinity hits against CK1α were obtained based on their binding affinity score. Furthermore, the ADMET, PAINS, and PASS evaluation was carried out to select more potent hits. Finally, following the interaction analysis, we elucidated three phytoconstituents, Semiglabrinol, Curcusone_A, and Liriodenine, posturing considerable affinity and specificity towards the CK1α binding pocket. The result was further evaluated by molecular dynamics (MD) simulations, dynamical cross-correlation matrix (DCCM), and principal components analysis (PCA), which revealed that binding of the selected compounds, especially Semiglabrinol, stabilizes CK1α and leads to fewer conformational fluctuations. The MM-PBSA analysis suggested an appreciable binding affinity of all three compounds toward CK1α.

## 1. Introduction

Casein kinase-1 alpha (CK1α) belongs to the serine/threonine family of kinases and functions primarily as a regulator in various signaling pathways [1]. Human kinases are considered as attractive drug targets for cancer therapy [2,3,4,5,6,7]. Like other kinases, CK1α performs multiple biological processes such as cell division, cell cycle, beta-catenin destruction and cell morphogenesis, signal transduction, WNT signaling pathway, etc. [8,9,10,11,12,13,14]. Pharmacological inhibition of CK1α has been investigated as a potential therapy in various diseases, including cancers [15]. In particular, CK1α controls the WNT signaling pathway, which is essential in driving hematologic malignancies [16]. It is considered an integral component of the WNT signaling or beta-catenin pathway and a potential drug target for cancer [1,17]. CK1α has shown high expression in various types of cancers like lymphoma, brain, prostate, lymphoma, and leukemia [1,18,19,20]. The RNA expression of CK1α is decreased in lung cancer, bladder cancer, and melanoma, which is in turn determined via the amount of protein expressed [1]. In progressed melanoma tumors, the downregulation of CK1α is mediated by its methylation [1]. 

**Figure 1:**
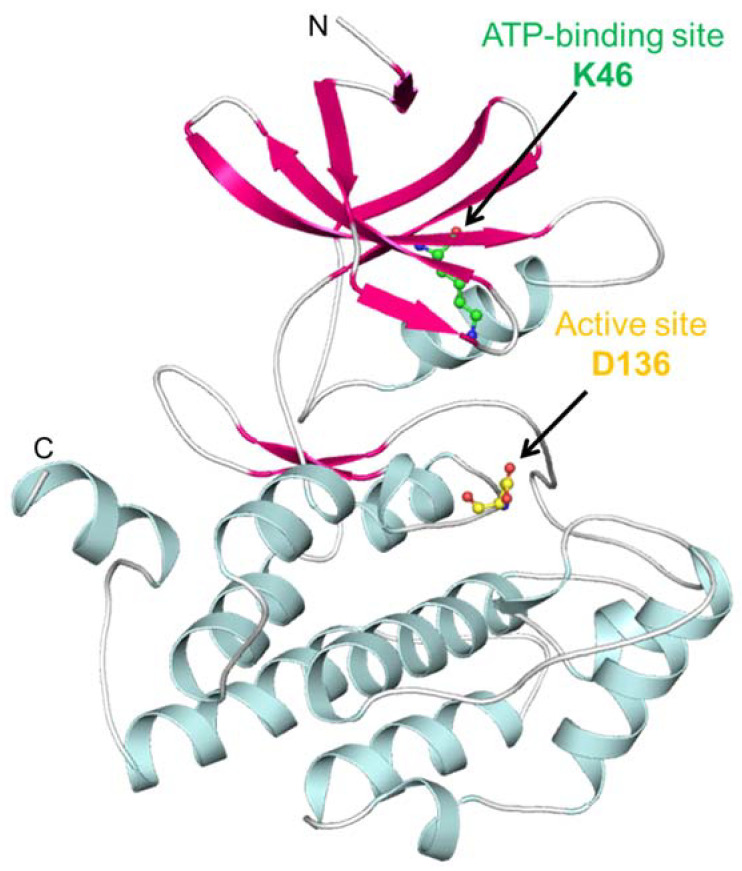
Structural features of Casein kinase 1α. Structural components, sheets, helices, and loops are shown in pink, cyan, and grey-white, respectively. The figure was drawn in PyMOL using PDB ID: 6GZD.

CK1 proteins contain an extremely conserved kinase domain at the N-terminal and a diverse regulatory domain at the C-terminus [21]. As a serine/threonine kinases family member, CK1α embodies the typical bilobal structure, consisting of beta-sheets at a smaller N-terminal lobe and an α-helical structure C-terminal lobe [22]. It consists of a conserved glycine-rich loop which forms the boundary of the ATP binding site and contributes the γ-phosphate moiety of ATP [22]. The ATP binding and active sites in CK1α are Lys46 and Asp136, respectively. The uniqueness around the binding pocket of CK1α facilitates its selective targeting for structure-based drug discovery. The structural features of CK1α are depicted in Figure 1.

Virtual screening has been an essential part of the drug discovery pipeline [23,24,25,26]. It helps find small molecules which could bind the defined target effectively and specifically [27,28,29,30,31,32]. It is one of the most effective techniques for identifying high-affinity binding partners to the target protein [23,24,25,33]. It computationally screens different chemical libraries available from various resources for identifying potential compounds [34,35,36,37]. The molecular docking-based virtual screening process, combined with several other filters, Lipinski’s filter [38], ADMET properties, PAINS filter [39], PASS analysis [40], carcinogenicity prediction, etc., accelerate the lead discovery process. Natural compounds, including phytoconstituents, have been considered an important source of leads in drug discovery for ages [41,42].

In this study, we have considered ~9500 phytoconstituents from the IMPPAT database, a curated database of phytochemicals of Indian medicinal plants. The compounds were subjected to screening based on Lipinski’s rule of five, followed by molecular docking, ADMET properties, and PASS evaluation [40]. From the top hits generated, we have further screened the compounds based on their specific interactions towards the CK1α binding pocket, followed by all-atom molecular dynamics (MD) simulations, dynamical cross-correlation matrix (DCCM), principal components analysis (PCA), and MM-PBSA analysis. Overall, the combined study suggests that three phytoconstituents, i.e., Curcusone A, Liriodenine, and Semiglabrinol targeting of CK1α, can be explored in the therapeutic management of cancer.

## 2. Materials and Methods

### 2.1. Computer Environment and Web Resources 

This study was performed on an HP Z840 workstation running on Windows 10 OS. We used high-speed internet with an uninterrupted power supply. Bioinformatics tools such as MGL AutoDock [43] and InstaDock [44] were used for molecular docking-based virtual screening; PyMOL [45] and Discovery Studio visualizers [46] were used for interaction analysis and visualization purposes. Various web-based servers and resources, including RCSB Protein Data Bank (PDB), IMPPAT (Indian Medicinal Plants, Phytochemistry, and Therapeutics) database [47], SwissADME [48], pkCSM [49], PASS [40], etc. were utilized for the retrieval, evaluation, and analyses purposes. 

### 2.2. Receptor Preparation and Library Preparation

The X-ray crystal structure of human CK1α (PDB ID: 6GZD, resolution: 2.28 Å) was downloaded from the RCSB-PDB in the PDB format and refined further using the InstaDock tool, New Delhi, India. Using the PyMOL, the structure was visualized, and water molecules and heteroatom, including co-crystallized ligand, were removed. A database named IMPPAT was used for the screening purpose. The Lipinski filter was applied to the IMPPAT database so that only compounds with admirable physicochemical properties were fetched out. IMPPAT is a manually curated database of traditional Indian medicines and other existing resources of phytochemistry. It has a total of 9596 compounds which remained to 5763 after applying the Lipinski filter.

### 2.3. Molecular Docking-Based Virtual Screening

Virtual screening plays a vital role in drug discovery and development [50,51]. It aims to screen large libraries of drug-like compounds computationally, generally commercially available, against specific protein targets and reduce them to a key set of likely drug candidates [52,53]. Molecular docking-based virtual screening is based on interacting receptors and small molecules [30,44,54,55]. The docking protocol tries to predict the position and orientation of the ligand when it is bound to a protein [56,57,58]. The docking process must be fast enough as many compounds are being analyzed [59,60,61,62]. In this attempt of virtual screening, we begin with a 3D structure of CK1α and a 3D database of phytoconstituents and score the compounds to identify lead candidates for further analysis. We used InstaDock to perform the molecular docking-based virtual screening. The docking screening was blind, where the search space was big enough to accommodate the protein’s entire structure and let the ligands freely move and search their favorable binding sites. The resultant output was analyzed in out-files and log-files once InstaDock completed the docking. The top hits were fetched out based on the affinity score toward CK1α.

### 2.4. ADMET Prediction

The filtered compounds from the docking results were subjected to filter out based on their ADMET properties. The prediction of ADMET properties along with PAINS (Pan-assay interference compounds) [39] evaluation was carried out using the pkCSM and SwissADME [48]. Compounds with well ADMET properties were taken and then filtered for any PAINS patterns. PAINS filter helps us to avoid compounds having explicit patterns with a higher tendency of binding to multiple targets [39]. The ADMET evaluation helps find compounds with drug-like physicochemical and pharmacokinetic properties, which reduces their chances of failure in clinical trials [63].

### 2.5. PASS Evaluation

The PASS analysis is useful in studying the chemical-biological interactions to evaluate the biological properties of chemical compounds. We have used the PASS server to examine the biological properties of the selected compounds from the ADMET filter. The internal algorithm of the PASS webserver uses molecular fragments of chemical compounds as multi-level neighbors of atoms descriptors and recommends certain biological properties. It provided two different descriptors, i.e., ‘probability to be active (Pa)’ and ‘probability of being inactive (Pi)’, where a higher Pa value signifies a higher probability of corresponding property for the compound.

### 2.6. Interaction Analysis

The interaction analysis of the docked protein-ligand complexes was performed to explore various interactions formed during their binding. The binding poses and all possible interactions were explored through the PyMOL and Discovery Studio Visualizer. The interactions formed within 3.5 Å within the protein-ligand complex were labeled as close contacts in the PyMOL. The type of interactions and the participating residual and atomic coordinates were explored through Discovery Studio Visualizer. Here, the compounds with specific interactions towards the critical residues of CK1α, including the active site and the binding site, were selected for further analyses. The binding of known CK1α binding partners was referred to compare the docking outputs. 

### 2.7. MD Simulations

#### 2.7.1. Systems Preparation and Simulation Protocol

The apo CK1α and its complexes with the selected ligands prepared through the molecular docking approach were used as initial coordinates in the MD simulation study. The all-atom MD simulations were performed through the AMBER 18 package [64]. The FF14SB AMBER force field [65] was used for the receptor protein, and appropriate charge and protonation state were prepared through the Protein Preparation Wizard implemented in the Schrödinger suite. The GAFF force field [66] was utilized for the ligands, and the AM1-BCC model [67] was used in parameterization and adding charges. The topology and atomic charges of the compounds were generated through the Antechamber utility of the AMBER 18 package. The topology and coordinates of the complex systems were generated through the leap module of AMBER 18. The solvation of all the systems was performed in a virtual box of the TIP3P water model [68]. An appropriate number of counterions was supplied for the neutralization of the systems. To deal with the hydrogen bonds and long-range electrostatic interactions, the SHAKE algorithm and the particle mesh Ewald (PME) was espoused, respectively. The energy minimization of all the systems was carried out using 10,000 steps of the steepest descent algorithm. Each system was gradually heated from 0 to 300 K for 100 ps. Afterward, the equilibration of each system was performed for 100 ps at 300 K and constant pressure. Lastly, a production run for 200 ns for each system was performed at constant temperature and pressure. The resultant trajectories were explored using the CPPTRAJ module [69]. The RMSD, RMSF, *R*_g_, SASA, H-bonds, secondary structure analysis, distance cross-correlation matrix, and PCA were analyzed from the generated outputs.

#### 2.7.2. Dynamical Cross-Correlation Matrix

The analysis of the MD resultant trajectory was also performed through the dynamical cross-correlation matrices (DCCM). DCCM analysis helps us to determine the coordinate aberrations and behaviors in C_α_ atoms of the protein [70]. In DCCM, all configurations are translated and rotated by the least-square-fitting method using all backbone C_α_ atoms of CK1α before and after the ligands binding to align on the equilibrated configurations. The technical concept of DCCM (*C_ij_*) is defined below:

(1) $$\;C_{ij}=\frac{\langle \Delta ri.\Delta rj\rangle }{{\left(\langle \Delta r_{i}^{2}\rangle \langle \Delta r_{j}^{2}\rangle \right)}^{\frac{1}{2}}}$$

where Δ*r_i,j_* signifies the ith and jth atom average point movement. Correlated movements are denoted by *C_ij_* = 1; however, *C_ij_* = −1 is supposed to be highly anti-correlated. The divergence of atomic movements from 1 to −1 defines that *i* and *j* movements are correlated and anti-correlated. 

#### 2.7.3. Principal Component Analysis 

Principal components analysis (PCA) is a highly useful approach in pattern recognition in protein movements [71]. In PCA, two-dimensional plotting of two different eigenvectors (EVs), i.e., EV1 and EV2, is produced by clustering them [27,33,72,73]. PCA was performed through the covariance matrix *C*, based on the atomic coordinates of C_α_ atoms and their corresponding eigenvalues [74]. The generation of positional covariance matrix *C* is defined below:

$$C_{i}=\langle \left(q_{i}-\langle q_{i}\rangle \right)\left(q_{j}-\langle q_{j}\rangle \right)\rangle \;\;\;\;\;\;\;\;\left(i,j=1,2,\ldots ,3N\right)$$

where *q_i_* and *q_j_* represent the cartesian coordinates for the *i*^th^, *j*^th^ position of the C_α_ atom and N is the number of C_α_ atoms.

#### 2.7.4. MM-PBSA Calculations 

To further support the binding studies of CK1 with the selected compounds, the binding affinity of each docked complex was examined through the MM-PBSA calculations [75]. The binding energies of each complex were estimated by considering the vacuum potential energy, including bonded and non-bonded interactions, and the free energy of solvation, considering polar and nonpolar terms. The polar solvation energy was calculated by resolving the Poisson-Boltzmann equation, while the nonpolar solvation energy was estimated using the SASA method. The MM-PBSA estimation was carried out while utilizing the script ‘MMPBSA.py’ of the AMBER suite [76]. 

## 3. Results and Discussion 

### 3.1. Molecular Docking-Based Virtual Screening

Molecular docking-based virtual screening of all the phytoconstituents from the IMPPAT was carried out to find high-affinity binding partners of CK1α. The resultant output generated the affinities and docked poses for each compound [30,54,77]. The compounds were filtered out based on their binding affinity towards CK1α. The selected compounds were found to possess appreciable binding affinity towards the binding pocket of CK1α (Table 1). The top 10 hits out of 5763 compounds had the binding affinity score ≤ −9.7 with CK1α (Table 1). The results indicated that the selected phytoconstituents have appreciable binding efficiency with CK1α, further exploring the therapeutic potential in the drug development process.

### 3.2. ADMET Properties

ADMET prediction consists of a set of parameters on which the pharmacokinetic properties of chemical compounds have to be depicted [29,30,78]. The selected hits from the docking study were further screened to predict their ADMET properties (Table 2). The three compounds out of 10 having ADMET within the range of drug candidacy were selected. These three compounds (Semiglabrinol, Curcusone_A, and Liriodenine) share a similar class of ADMET properties without any toxic patterns (AMES/Hepatotoxicity) thus were selected for further analysis. 

### 3.3. PASS Evaluation

Natural compounds possess many chemico-biological properties, which may consequence in synergistic or antagonistic impacts [41,79]. In search of safe and effective compounds with desirable properties, the biological properties for the hit molecules need to be explored. In this study, the PASS analysis explored the probable properties of the elucidated hits. The compounds and their biological properties are summarized in Table 3, along with their confidence level. The results revealed that the selected hits, Semiglabrinol, Curcusone_A, and Liriodenine, possess antineoplastic and kinase inhibitory potential, with significant Pa values, i.e., 0.612 to 0.889. The PASS analysis recommended that Curcusone_A, Liriodenine, and Semiglabrinol have great potential in anticancer therapeutics.

### 3.4. Interaction Analysis 

**Figure 2:**
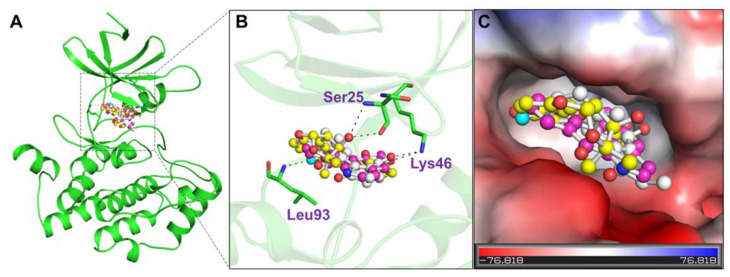
Molecular interactions of (**A**) Casein kinase 1 with Curcusone A (white), Liriodenine (magenta), and Semiglabrinol (yellow). (**B**) Magnified cartoon view of protein-ligands interactions. (**C**) Electrostatic potential of Casein kinase 1 bound the selected compounds.

The selected compounds’ binding modes and interaction patterns were analyzed utilizing PyMOL and Discovery Studio visualizer. The analysis of compounds was done based on interacting residues. We employed Discovery Studio and PyMOL to identify and visualize hydrogen bonding and other interactions of the compounds with CK1α. It was found that residues of the kinase domain of CK1α offer a significant number of interactions, such as Ser25, Lys46, and Leu93 (Figure 2). The ATP binding site, i.e., Lys46, was also found to make direct contact with the docked compounds, which is crucial for the CK1α activity (Figure 2B). All three compounds were found to be fit within the binding pocket of CK1α with a good complementarity (Figure 2C). 

**Figure 3:**
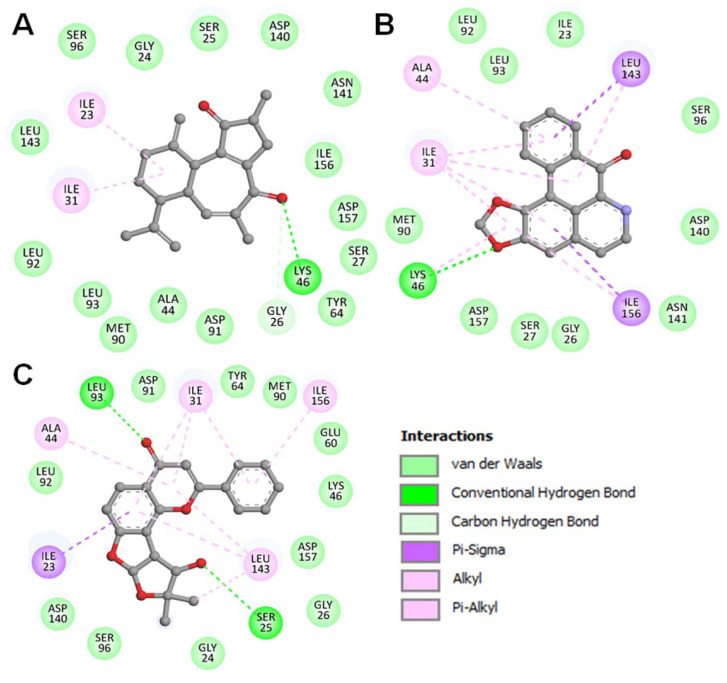
Representation of Molecular Interaction and 2D plots showing detailed interactions of (**A**) Curcusone A, (**B**) Liriodenine, and (**C**) Semiglabrinol with CK1α.

The detailed binding analysis showed that the interaction of all three compounds was in the ATP binding pocket, where several crucial residues of CK1α participated in the interaction (Figure 3). The binding of all three compounds with CK1α was stabilized by several interactions, including four conventional H-bonds, one carbon-H bond, and a few hydrophobic interactions. The plot showed that two H-bonds stabilized the CK1α-Curcusone_A complex with Gly26 and Lys46, two Alkyl bonds with Ile23 and Ile31, along with 15 Van der Waals interactions (Figure 3A). While one H-bond stabilized the CK1α-Liriodenine complex with Lys46, two Alkyl bonds with Ile31 and Ala44, along with two Pi-sigma bonds with Leu143 and Ile156, and 10 Van der Waals interactions (Figure 3B). At the same time, the CK1α-Semiglabrinol complex was stabilized by two H-bonds with Ser25 and Ile93, along with several other interactions (Figure 3C). The stable binding of the elucidated compounds with the ATP-binding site might be vital to inhibit the kinase activity of CK1α and raise them as “competitive inhibitors” of CK1α. 

### 3.5. MD Simulations

#### 3.5.1. Structural Deviations in CK1α

**Figure 4:**
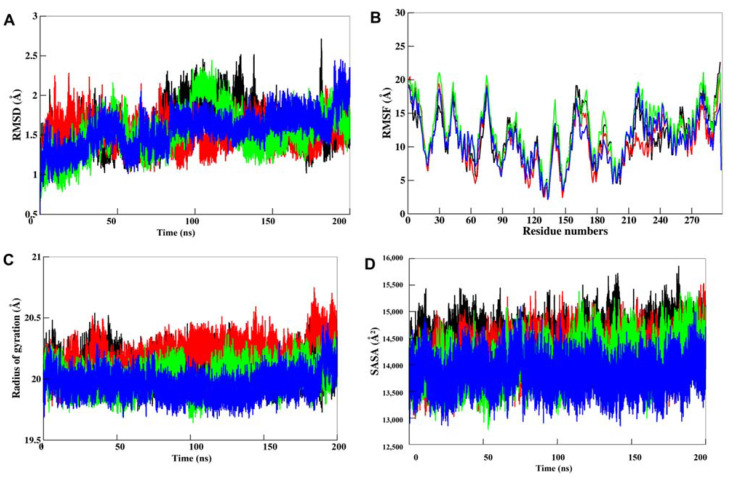
Structural dynamics of CK1α Apo (black), CK1α-Curcusone_A (red), CK1α-Liriodenine (green), and CK1α-Semiglabrinol (blue) (**A**) RMSD, (**B**) RMSF, (**C**) Rg, and (**D**) SASA across Cα backbone calculated after 200 ns of MD trajectories.

Docking study can only provide a static prototype for protein-ligand interactions; hence, MD simulation studies were carried out on CK1α and its docked complexes with Curcusone_A, Liriodenine, and Semiglabrinol to explore their binding mechanism. To assess the structural deviations in CK1α and its docked complexes, the systematic properties of each complex, such as RMSD and RMSF, were examined during the simulation time. The fluctuations of RMSD values in each system are depicted in Figure 4A, which indicates that all of them are stable without any major fluctuation during the 200 ns MD trajectories. The RMSD values of Cα backbone atoms denoting the starting structure were used to observe the dynamic stability of the complexes. The RMSD results revealed that each complex reached equilibrium after 50 ns which are quite stable up to the simulation trajectory.

To evaluate the stability of the binding pocket residues in MD simulation, the RMSF of all the residues in CK1α were calculated and plotted [80,81,82,83,84]. During the RMSF calculation, the average fluctuation of each residue of CK1α before and after the binding of the compounds was computed for the entire 200 ns trajectories of MD simulation. The RMSF results showed that residues around the ligand-binding site are less fluctuated than other regions, suggesting that the binding pocket is relatively stable during the simulation time (Figure 4B). 

The *R_g_* is another useful tool to explore the compactness of a protein and protein-ligand complex [53,85]. A higher *R_g_* value for a protein indicates its loose packing, while; lower *R_g_* value indicates tight packing of the structure. In MD studies, it is used to demonstrate the impact of a ligand molecule, exerting conformational changes in protein molecules. We have evaluated the *R_g_* of each complex during the simulation to see the impact of compounds binding on the conformational packing of CK1α (Figure 4C). The results showed that CK1α had the lowest *R_g_* value during the simulation when in complexed with Semiglabrinol, which could be attributed to a more compact structure than the free state of CK1α. However, the results indicated that the ligand binding to CK1α doesn’t affect its compactness and supports complex stability. 

To further evaluate the folding/unfolding behavior of CK1α before and ligand binding, we have calculated the time evolution of SASA values during the simulation. The SASA values represent the exposure of each amino acid in a protein surrounded by the solvent. The plotted SASA values are in good agreement with the *R_g_* trend of the simulated trajectories of 200 ns (Figure 4D). The plot showed that SASA is decreased after the binding of a ligand, especially in the case of Semiglabrinol, which suggested higher compactness and stability of the docked complexes during the simulation. After the 100 ns simulation, the SASA values of the CK1α-Liriodenin rise up slightly in the rest of the simulation time, which indicated an exposer of some buried residues to the solvent, but without any structural shift. 

#### 3.5.2. Dynamics of Hydrogen Bonds 

**Figure 5:**
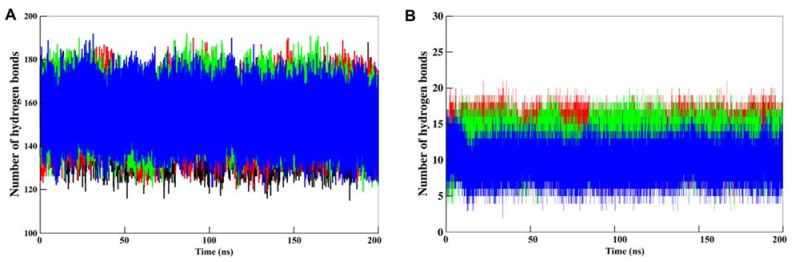
The dynamics of H-bonds in CK1α. (**A**) Intramolecular and (**B**) Intermolecular hydrogen bond analysis in CK1-Curcusone_A (red), CK1-Liriodenine (green), and CK1-Semiglabrinol (blue) calculated after 200 ns MD simulation.

Intramolecular H-bonds formation is quite important in maintaining the integrity of protein structure [86]. The analysis of intramolecular H-bonds during MD simulation is useful to examine the impact of ligand binding on the protein structure. At the same time, the analysis of intermolecular H-bonds is useful to see the lifetime of interactions formed between the protein and ligand. This work monitored the time evolution of H-bonds, with the distance cutoff set to 3.5 Å. The resultant plots of intramolecular and intermolecular H-bonds analysis for all the four systems in MD simulations are shown in Figure 5. The generated plot showed an overlapped pattern of intermolecular H-bonds distribution that suggested that CK1a maintained its structural integrity during the simulation even after compounds binding (Figure 5A). This analysis of intermolecular H-bonding also indicated that the compounds haven’t moved from their initial docking position on CK1α and maintain the interactions in stabilizing the complex structures (Figure 5B).

#### 3.5.3. Secondary Structure Dynamics

**Figure 6:**
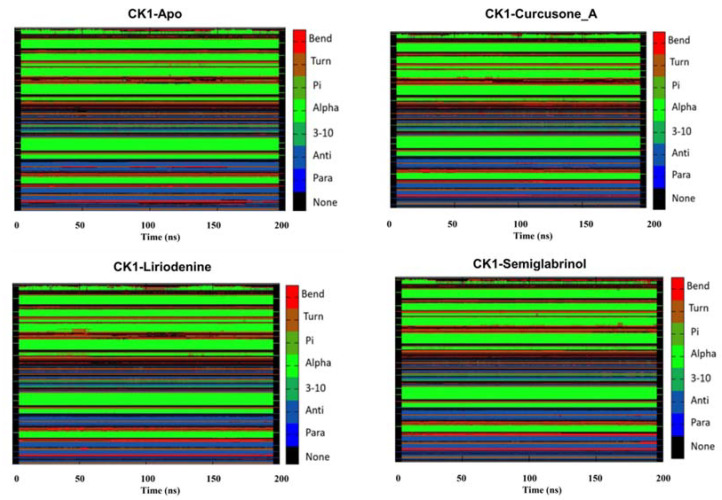
Secondary structural analysis of CK1α Apo, CK1α-Curcusone_A, CK1α-Liriodenine and CK1α-Semiglabrinol complexes calculated after 200 ns of MD trajectories.

Secondary structure components in a protein maintained its 3D structure and regulated the flexibility/rigidity of the protein in a natural system [87,88,89,90]. To see the impact of compounds binding on CK1α, the dynamics of secondary structure components were monitored (Figure 6). The generated graphs show the participation of each residue in secondary structure formation overtime during the entire simulation. The secondary structure panels of each system generated through the simulated trajectory indicated the stable pattern over 200 ns simulation. The result suggested that secondary structure components of CK1α protein are conserved before and after the binding of each compound during the entire simulation. The average number of residues participating in the secondary structure formation is summarized in (Table 4). 

However, a slight decrement in α-helices and β-sheets in the case of CK1α-Curcusone_A was observed, possibly due to its increased dynamics as seen in the RMSF and SASA analyses. Whereas, a slight increase in the average number of residues that participated in the formation of α-helices and β-sheets of CK1α was observed after the Semiglabrinol binding, which suggested more compactness and stability of the docked complex during the simulation. 

#### 3.5.4. DCCM

**Figure 7:**
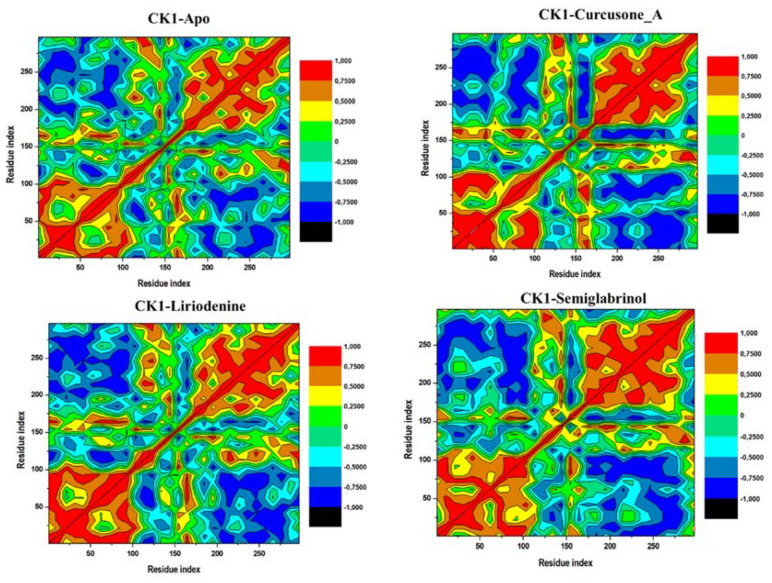
Dynamical cross-correlation matrices of the Apo CK1α, CK1α-Curcusone_A, CK1α-Liriodenine and CK1α-Semiglabrinol complexes.

Motions in a protein take place on a comprehensive range of time scales, extending from femtoseconds to seconds [91]. DCCM also depends on the time scale over which the correlation data was composed [92]. To explore the correlated and anti-correlated movements in CK1α and its docked complexes with Curcusone_A, Liriodenine, and CK1α-Semiglabrinol, the inter-residue DCCM analysis was carried out. Four DCCM plots generated for the CK1α and its docked complexes are illustrated in Figure 7. Positive and negative correlations are shown in red and blue, respectively. The maps indicated that CK1α scattered into different populations through positive and negative correlations with reference to the residue index. The movements in all maps were relatively alike with minor fluctuations, suggesting that CK1α may have similar global dynamics before and after compound binding. Overall, the correlation patterns in all the graphs are weakly differentiated, suggesting the stability of the movements in free CK1α and its ligand-bound complexes.

#### 3.5.5. PCA 

**Figure 8:**
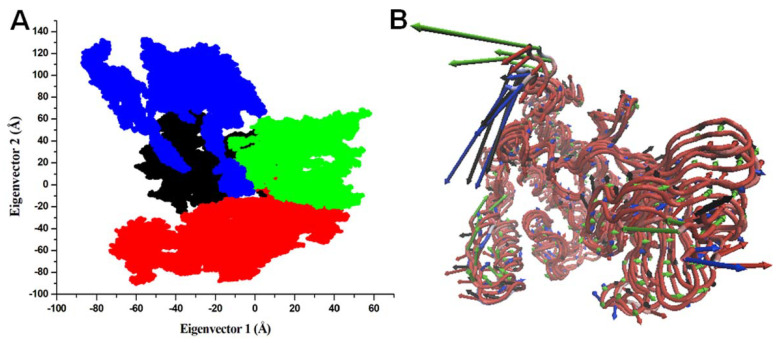
Principal component analysis. (**A**) 2D projection of CK1α Apo (black), CK1α-Curcusone_A (red), CK1α-Liriodenine (green), and CK1α-Semiglabrinol complex calculated after 200 ns of MD trajectories (bule). (**B**) PC1 collective motions in porcupine plot for Apo, Curcusone_A, Liriodenine, and Semiglabrinol CK1α complexes.

PCA is a useful approach in figuring out the overall combined motions of the Cα atoms in a protein represented by the EVs of the covariance matrix [93]. It is used to explore the collective motions and conformational sampling of a protein and protein-ligand complex [94,95]. Employing PCA has been a valuable approach to studying the folding dynamics of a protein in the presence of small molecules [26,96,97]. To further explore the directionality of the conformational motions in CK1α, we carried out the PCA of all four simulation trajectories (Apo CK1α, CK1α-Curcusone_A, CK1α-Liriodenine, and CK1α-Semiglabrinol) (Figure 8). The average of the protein motions was designated based on the Cα atoms of CK1α. The projections conformations for the first two EVs, EV1, and EV2 indicated that CK1α in complexed with Curcusone_A has significantly higher negative motions than the apo CK1α and its other complexes. The results suggested CK1α-Semiglabrinol higher stable that mimicked the motions of the apo CK1α with some positive movements. In all four projections, the CK1α-Curcusone_A and CK1α-Liriodenine complexes occupied a broader phase space as compared to the Apo CK1α and CK1α-Semiglabrinol complex (Figure 8A). The collective motions of the CK1α-Semiglabrinol complex may plausibly enhance the stability of the docked complex compared to others. The directionality and magnitudes of all four trajectories were also explored through porcupine plots (Figure 8B). The changes in direction and magnitude of the complexed systems indicated that the binding of the identified compounds induced a minor conformational impact on the conformational dynamics of CK1α but a quite stabilization during the simulation. 

#### 3.5.6. MMPBSA

The solvent condition is one of the crucial parameters while determining the binding affinity of a ligand molecule with a protein, which is typically not considered in molecular docking studies [98]. Therefore, the binding energies of all three docked complexes were further obtained through MM-PBSA calculations using the simulated MD trajectories. The binding energies of all three docked complexes are summarized in Table 5**.** The calculated binding energy through the MM-PBSA approach includes different energetic terms for the bonded and non-bonded (van der Waals and electrostatic) interactions. The MM-PBSA calculation was performed on the last 50 ns of the production run. The calculated results showed that Semiglabrinol in complex with CK1α has the best result in MM-PBSA binding energy, further suggesting their higher stability than others. MM-PBSA result showed that the docked complex of Semiglabrinol with CK1α was stabled with a binding affinity (Δ*G*) −41.08 kcal/mol. The negative value of ∆E_ele_ as compared to ∆E_vdW_ and ∆*G*_nonpolar_ signified the presence of H-bonds and polar interactions between the CK1α and the ligands, especially Semiglabrinol. Overall, the MMPBSA analysis confirmed the stability of all the docked complexes.

## 4. Conclusions

Considering CK1α as a potential therapeutic target because of its presence as a positive regulator of cancer progression and targeting it with the selected hits is an attractive strategy. The computational approach used in this study might prove its usefulness in developing potential leads from natural compounds as potent inhibitors of CK1α against cancer. We performed a systematic study of the structure-based drug discovery approach and identified three phytoconstituents, Semiglabrinol, Curcusone_A, and Liriodenine, evaluated as potent binding partners of CK1α. Initially, the compound database was filtered out based on several drug-like properties, followed by molecular docking study. Then, the results were validated by investigating RMSD, RMSF, *R_g_*, SASA, and intra- and intermolecular H-bonding analyses in MD simulations followed by DCCM, and PCA. The integrated approach was used to assure that the selected hits interact properly with CK1α with considerable stability. The MM-PBSA analysis further indicates that the elucidated phytoconstituents can act as promising CK1α inhibitors and further be exploited for drug design purposes.

## Figures and Tables

**Table 1:** The top 10 hits and their binding affinities toward CK1α.

S. No.	Compound ID	Phytochemical Name	Source	Binding Affinity (kcal/mol)	pKi	* Ligand Efficiency
1.	443716	Hydroxysanguinarine	Papaver somniferum	−10.1	7.41	0.33
2.	94577	Cepharadione A	Piper nigrum	−10.0	7.33	0.33
3.	11035494	Semiglabrinol	Tephrosia purpurea	−9.9	7.26	0.30
4.	124069	Dihydrosanguinarine	Fumaria indica	−9.8	7.19	0.33
5.	175941	Curcusone A	Jatropha curcas	−9.8	7.19	0.39
6.	10144	Liriodenine	Annona squamosa	−9.8	7.19	0.38
7.	147329	Corysamine	Meconopsis aculeata	−9.7	7.11	0.33
8.	197018	Ushinsunine	Michelia champaca	−9.7	7.11	0.31
9.	2754650	Irenolone	Musa paradisiaca	−9.7	7.11	0.35
10.	442851	Crinasiatine	Crinum asiaticum	−9.7	7.11	0.33

* Ligand Efficiency values are in (kcal/mol/non-H atom); S. No., serial number.

**Table 2:** ADMET properties of the top 10 compounds.

S. No.	Compound	Absorption		Distribution	Metabolism	Excretion	Toxicity
		*GI* *Absorption*	*Water* *Solubility*	*BBB* *Permeation*	*CYP2D6* *Substrate/Inhibitor*	*OCT2* *Substrate*	*AMES/Hepatotoxicity*
1.	Hydroxysanguinarine	High	Moderate	Yes	No	No	Yes
2.	Cepharadione A	High	Moderate	Yes	No	No	Yes
3.	Semiglabrinol	High	Moderate	Yes	No	No	No
4.	Dihydrosanguinarine	High	Moderate	Yes	No	No	Yes
5.	Curcusone A	High	Moderate	Yes	No	Yes	No
6.	Liriodenine	High	Moderate	Yes	Yes	No	No
7.	Corysamine	High	Moderate	Yes	Yes	Yes	Yes
8.	Ushinsunine	High	High	Yes	Yes	No	Yes
9.	Irenolone	High	Moderate	Yes	Yes	No	Yes
10.	Crinasiatine	High	Moderate	Yes	Yes	No	Yes

S. No., serial number; GI absorption, gastrointestinal absorption; BBB permeation, blood-brain barrier permeation.

**Table 3:** Biological properties of the elucidated phytoconstituents predicted through the PASS webserver.

**Compound ID**	**Pa**	**Pi**	**Biological Activity**
Semiglabrino	0.808	0.005	Kinase inhibitor
	0.793	0.011	Membrane permeability inhibitor
	0.783	0.014	Antineoplastic
	0.653	0.036	TP53 expression enhancer
	0.612	0.033	Oxidoreductase inhibitor
Curcusone_A	0.889	0.005	Antineoplastic
	0.819	0.015	Antieczematic
	0.803	0.004	Carminative
	0.744	0.004	Transcription factor NF kappa B stimulant
	0.702	0.015	Apoptosis agonist
Liriodenine	0.784	0.014	Antineoplastic
	0.763	0.008	Caspase 3 stimulant
	0.710	0.015	Alkane 1-monooxygenase inhibitor
	0.680	0.014	Kinase inhibitor
	0.629	0.005	Caspase 8 stimulant

**Table 4:** Percentage of amino acid residues contributed to the secondary structure of CK1α Apo, CK1α-Curcusone_A, CK1α-Liriodenine and CK1α-Semiglabrinol complexes calculated after 200 ns of MD trajectories.

Complex	α	β	3_10_-Helix	Turn	Bend	Other
CK1α-Apo	24	23	3	9	11	19
CK1α-Curcusone_A	21	19	5	10	8	21
CK1α-Liriodenine	27	23	6	12	10	18
CK1α-Semiglabrinol	28	26	7	13	14	24

**Table 5:** MM-GBSA energy profiles of CK1α in complex with Curcusone_A, Liriodenine, and Semiglabrinol *.

Complex	∆EvdW	∆Eele	∆Ggas	∆Gpolar	∆Gnonpolar	∆Gsol	∆Gbind
CK1α-Curcusone_A	−45.23	−8.27	−53.50	20.77	−5.74	15.03	−38.47
CK1α-Liriodenine	−38.60	−9.31	−47.91	20.77	−4.24	16.53	−31.38
CK1α-Semiglabrinol	−43.58	−1.98	−45.56	9.54	−5.06	4.47	−41.08

* All the values are in kcal/mol.
